# A comparative analysis of RNA sequencing methods with ribosome RNA depletion for degraded and low-input total RNA from formalin-fixed and paraffin-embedded samples

**DOI:** 10.1186/s12864-019-6166-3

**Published:** 2019-11-08

**Authors:** Xiaojing Lin, Lihong Qiu, Xue Song, Junyan Hou, Weizhi Chen, Jun Zhao

**Affiliations:** 1 Department of Thoracic Surgery, Cancer Hospital Chinese Academy of Medical Sciences & Peking Union Medical College, Beijing, China; 2 Genecast Precision Medicine Technology Institute, Room 903-908, Health work, Huayuan North Road 35, Haidian District, Beijing, 100191 China

**Keywords:** RNA-seq, rRNA depletion, HISAT, Degraded FFPE sample

## Abstract

**Background:**

Formalin-fixed and paraffin-embedded (FFPE) blocks held in clinical laboratories are an invaluable resource for clinical research, especially in the era of personalized medicine. It is important to accurately quantitate gene expression with degraded and small amounts of total RNA from FFPE materials.

**Results:**

High concordance in transcript quantifications were shown between FF and FFPE samples using the same kit. The gene expression using the TaKaRa kit showed a difference with other kits, which may be due to the different principle of rRNA depletion or the amount of input total RNA. For seriously degraded RNA from FFPE samples, libraries could be constructed with as low as 50 ng of total RNA, although there was residual rRNA in the libraries. Data analysis with HISAT demonstrated that the unique mapping ratio, percentage of exons in unique mapping reads and number of detected genes decreased along with the decreasing quality of input RNA.

**Conclusions:**

The method of RNA library construction with rRNA depletion can be used for clinical FFPE samples. For degraded and low-input RNA samples, it is still possible to obtain repeatable RNA expression profiling but with a low unique mapping ratio and high residual rRNA.

## Background

With the development of massive parallel sequencing, RNA-Seq has become an useful tool for transcriptome analysis, as well as for the identification of novel transcripts, SNPs, gene fusion and alternative splicing events [1]. Formalin-fixed and paraffin-embedded (FFPE) blocks held in clinical laboratories are an invaluable resource for clinical research, especially in the era of personalized medicine. FFPE samples are easy to store, preserve tissue morphology for clinical and pathological observation, and preserve nucleic acids for molecular biology research [2]. Currently, many clinical tests are based on the expression of certain genes, such as the MammaPrint test, to assess recurrence risk in early-stage breast cancer [3] and the tissue of origin (TOO) test to find the site of the primary tumor. In addition, RNA expression profiles have become an important source of new biomarkers with potential values in cancer metastasis and disease prognosis [4, 5]. The discovery and development of these diagnostic and prognostic biomarkers will rely heavily on retrospective studies on historical FFPE samples [6]. Therefore, it is important to accurately quantitate the gene expression with total RNA from FFPE materials.

RNA-seq requires the enrichment of mature mRNAs, or the depletion of highly abundant ribosomal RNAs (rRNAs) from total RNA before sequencing. RNAs from FFPE materials are usually degraded to small sizes without the 3′poly (A) tail; moreover, recent studies suggest that certain functionally important mRNAs are non-poly (A) RNAs [7]. Therefore, capturing the 3′poly (A) tail is not a compatible method, especially when the starting materials are from FFPE samples. Another method for RNA-seq of FFPE samples is cDNA hybrid capture using a whole exome DNA probe to hybridize to the total RNA library. The yield of on-exon data was increased significantly due to the cDNA-capture, while the accuracy of quantitated gene expression was decreased [8, 9]. The signals of low gene expression might be missed by decreased uniformity of the exome probe.

For RNA-seq of FFPE samples, rRNA depletion from total RNA is the optimal solution. Nucleic acids extracted from FFPE blocks are fragmented and chemically modified, making them controversial to use in molecular diagnosis. rRNA depletion protocols could keep as much information as possible from the total RNA. There are several rRNA depletion protocols. The first method that is commonly used hybridizes the rRNA to a DNA probe and degrades the rRNA: DNA hybrids using RNase H. In the second method, rRNA is captured by complementary DNAs, which are coupled to paramagnetic beads, and the mixture is removed from the reaction [10]. Several studies have shown that FFPE RNA-seq data produced high concordance with RNA-seq results from matched frozen fresh samples [11, 12]. Previous studies have confirmed that for low-quality RNA, especially for degraded FFPE RNA, the RNase H method performed best [13]. The third method, which is suitable for low-input and low-quality samples, first transcribes total RNA to cDNA, and then the ZapR enzyme digests all rRNA: DNA hybrids. With an increasing number of commercially available RNA library preparation kits based on the principle of rRNA removal, we can make the best use of clinical FFPE samples. All those kits utilizing these methods are available, but the effect of the efficiency of rRNA removal on RNA-seq data is still unclear.

**Fig. 1:**
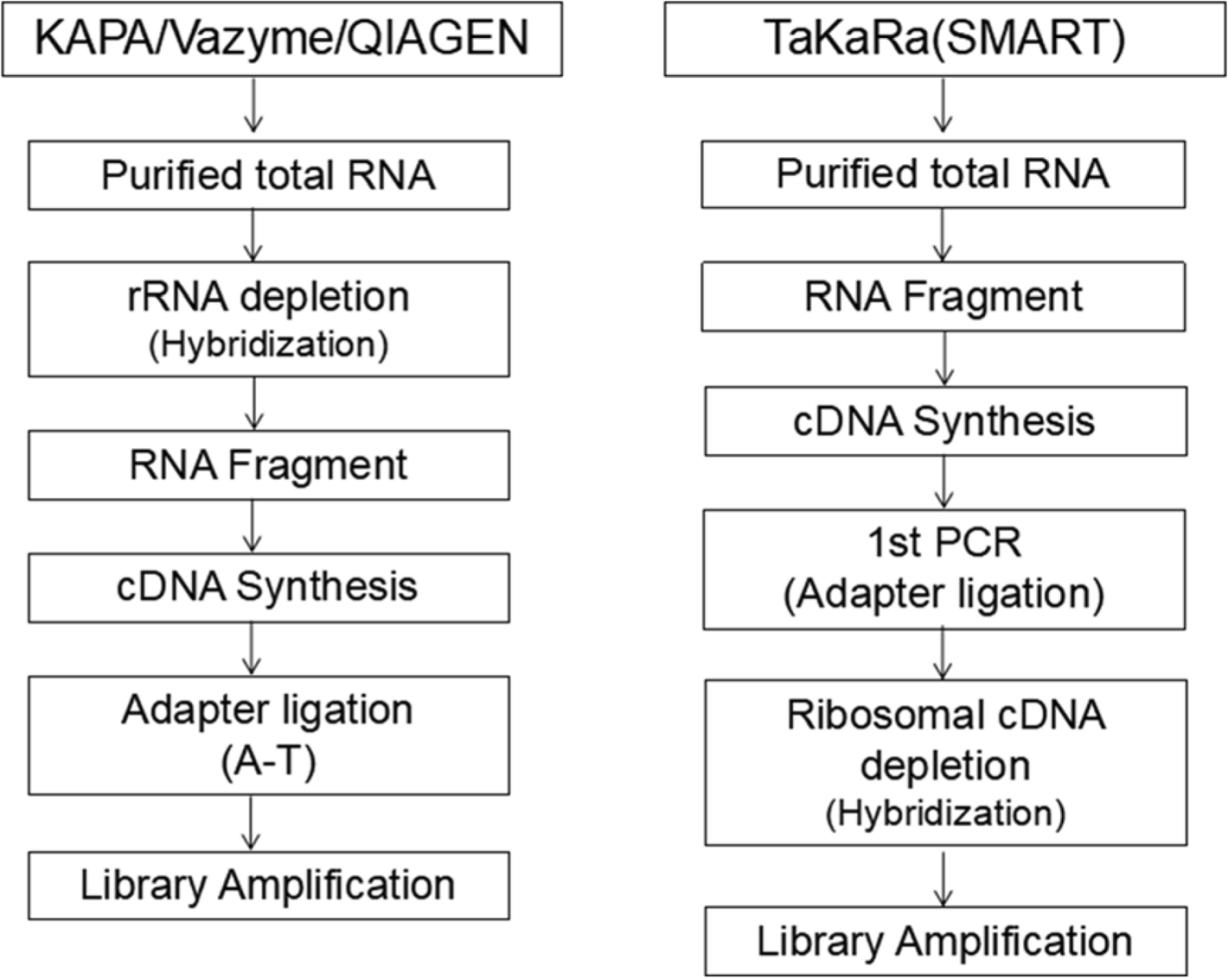
Schematic overview of four RNA-seq library preparation kits based on rRNA removal protocols

In this study, we compared four FFPE RNA library preparation kits (KAPA, TaKaRa, QIAGEN and Vazyme) based on two principles of rRNA depletion, with degraded RNA from FFPE samples and paired FF samples as starting materials (Fig. 1). Takara Kit only requires input of 5 to 50 ng total RNA with chemical modifications, such as those extracted from FFPE tissue and input of 250 pg to 10 ng total RNA for FF samples. After total RNA was fragmented or denatured, cDNA was synthesized, including cDNA from rRNA. In the next step, the synthesis of cDNA was added full-length Illumina adapters by a first round of PCR amplification (PCR1), including barcodes. And then, originating from rRNA of the ribosomal cDNA was cleaved by ZapR in the presence of the R-Probes. Finally, untouched and originating from non-rRNA molecules were enriched by a second round of PCR amplification (PCR2), and purified the final library.

KAPA kit has been validated for library construction from 25 ng to 1 μg of total RNA. This kit using Oligo Hybridization and rRNA Depletion eliminated the effect of ribosomal RNA on library. The rRNA duplexed to DNA oligos was digested by RNase H treatment. Before the cDNA synthesis, hybridization oligos were removed from the sample by DNase I digestion. The rRNA-depleted RNA is eluted and fragmented to the desired size using high temperature in the presence of Mg^2+^. And then, 1st strand and 2nd strand cDNA was synthesized successively, of which 2nd strand cDNA was marked by dUTP. The dAMP was then added to the 3′-end of dscDNA fragments, and 3′-dTMP adapters are ligated to 3′-dAMP library fragments. After fragment separation, PCR amplification was performed on the final library.

Vazyme kit is mainly applicable to the total RNA of human, mouse and rat with a starting value of 0.1–1 μg, and also applicable to the construction of the library for the degradation of RNA samples of the above species. QIAGEN Kit need 1–100 ng enriched, poly(A)^ +^ RNA. So we used the first few steps of Vaths™ Total RNA-seq (H/M/R) Library Prep Kit protocol to get the poly(A) + RNA. The removal of ribosomal RNA from both Vazyme and QIAGEN kits was similar to KAPA kit.

In addition, we evaluated the effect of bioanalysis tools on the total mapping rate, unique mapping rate, exon percentage and number of detected genes using FF samples and FFPE samples. HISAT (hierarchical indexing for spliced alignment of transcripts) allows scientists to align reads to a genome, assemble transcripts, compute the abundance of these transcripts in each sample and compare experiments to identify differentially expressed genes and transcripts [14]. STAR (Spliced Transcripts Alignment to a Reference) can discover noncanonical splices and chimeric (fusion) transcripts and is also capable of mapping full-length RNA sequences [15]. STAR generates output files that can be used for many downstream analyses, such as transcript/gene expression quantification, differential gene expression, novel isoform reconstruction, signal visualization, and so forth [16]. Both tools are free, open-source methods for comprehensive analysis of RNA-seq experiments.

In the last part of this study we evaluated the performance of two kits allowing for lower input of total RNA because many clinical studies need to use RNA, even though a low quality and a very low input of RNA can be extracted from clinical FFPE samples. We also validated the reproducibility of low-quality and low-quantity samples.

## Results

### Performance of four RNA-seq preparation kits for FF and FFPE samples

To evaluate the performance of four RNA-seq preparation kits, we collected total RNA from GM12878 FF and FFPE samples. The quality of the two RNA samples is shown in Additional file 1: Figure S1. We constructed RNA-seq libraries following the recommended protocols respectively. After sequencing, the raw data of all eight libraries were down sampled to 18 G and analytical comparisons were focused on several fields including the yield of libraries, GC content, rRNA depletion efficiency, genome alignment profiles, transcriptome coverage, transcript quantification, etc. (Table 1).

**Table 1:** Comparison of four RNA library preparation kits for FFPE and FF samples

Kits	KAPA		TaKaRa		Vazyme		QIAGEN	
Sample	FFPE	FF	FFPE	FF	FFPE	FF	FFPE	FF
Recommended input	25 ng-1 μg		5–50 ng	0.25–10 ng	100 ng-1 μg		100 ng-5 μg	
Input total RNA (ng)	100	100	10	10	100	100	100	100
PCR cycles	15	15	16	13	15	15	15	15
Library (ng)	178.4	1048.0	792.0	944.0	317.5	945.0	196.8	408.0
Total raw data (G)	35.7	33.6	21.7	18.1	35.4	42.2	36.8	31.2
Downsampled data (G)	18.0	18.0	18.0	18.0	18.0	18.0	18.0	18.0
Clean bases (G)	16.4	16.2	14.0	14.0	15.8	15.6	16.8	16.9
rRNA (%)	1.46	1.29	13.47	12.77	1.20	0.82	0.72	0.54
Q30 (%)	90.84	90.49	93.98	93.91	90.42	90.15	92.69	92.60
GC (%)	53.25	55.87	53.02	53.90	49.22	52.05	49.58	50.82
Total mapping rate (%)	96.32	96.41	91.63	93.90	95.38	94.84	97.36	97.46
Unique mapping rate (%)	80.90	79.10	79.33	80.61	84.56	81.66	85.54	84.49
Multiple mapping rate (%)	15.42	17.31	12.30	13.29	10.82	13.18	11.82	12.97
Exon (%)	53.39	75.06	64.78	70.25	44.87	67.21	48.50	70.46
Intron (%)	44.52	23.12	33.55	26.87	52.95	30.81	49.47	27.92
Intergenic (%)	2.09	1.82	1.68	2.88	2.18	1.98	2.03	1.62
Transcript (FPKM > = 0.3)	23,749	22,099	22,046	32,221	24,420	22,718	23,788	22,397
Transcript (FPKM > = 1)	18,667	16,255	16,782	18,079	19,501	17,247	18,892	16,712

**Fig. 2:**
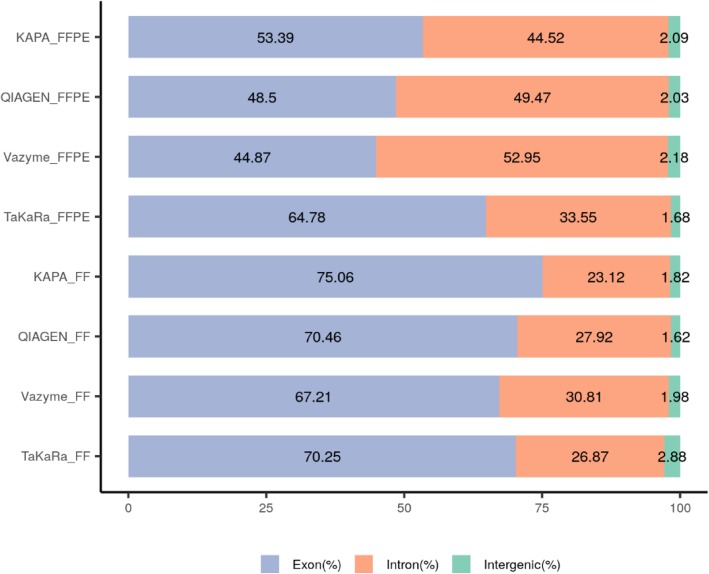
Genome alignment profiles of four RNA-seq kits with paired FFPE and FF samples. For FF RNA from GM 12878 cell line, all the four kits got similar alignment profiles while the input RNA of TaKaRa kit was 10 ng and it of the others was 100 ng. For FFPE RNA from GM 12878 cell line, the library with TaKaRa kit produced more exon profiles with 10 ng total RNA input

The recommended input is even lower for the TaKaRa kit than the other three kits, so we input 10 ng of total RNA for preparing the library, while the input of the other kits was 100 ng. The library yields and exon percent in the unique mapping data of the FFPE sample with the TaKaRa kit was the highest (Table 1 and Figure 2), which indicated that the TaKaRa kit is intended for low-input starting material. The performance of the other three kits showed a similar tendency of the library yields and exon percentage in the unique mapping data of the FFPE samples being much lower than that of the FF samples. Residual rRNA in the TaKaRa library was also the highest and had the least clean data, which was due to the removal of ribosomal cDNA (cDNA fragments originating from rRNA molecules) after cDNA synthesis using probes specific to mammalian rRNA.

**Fig. 3:**
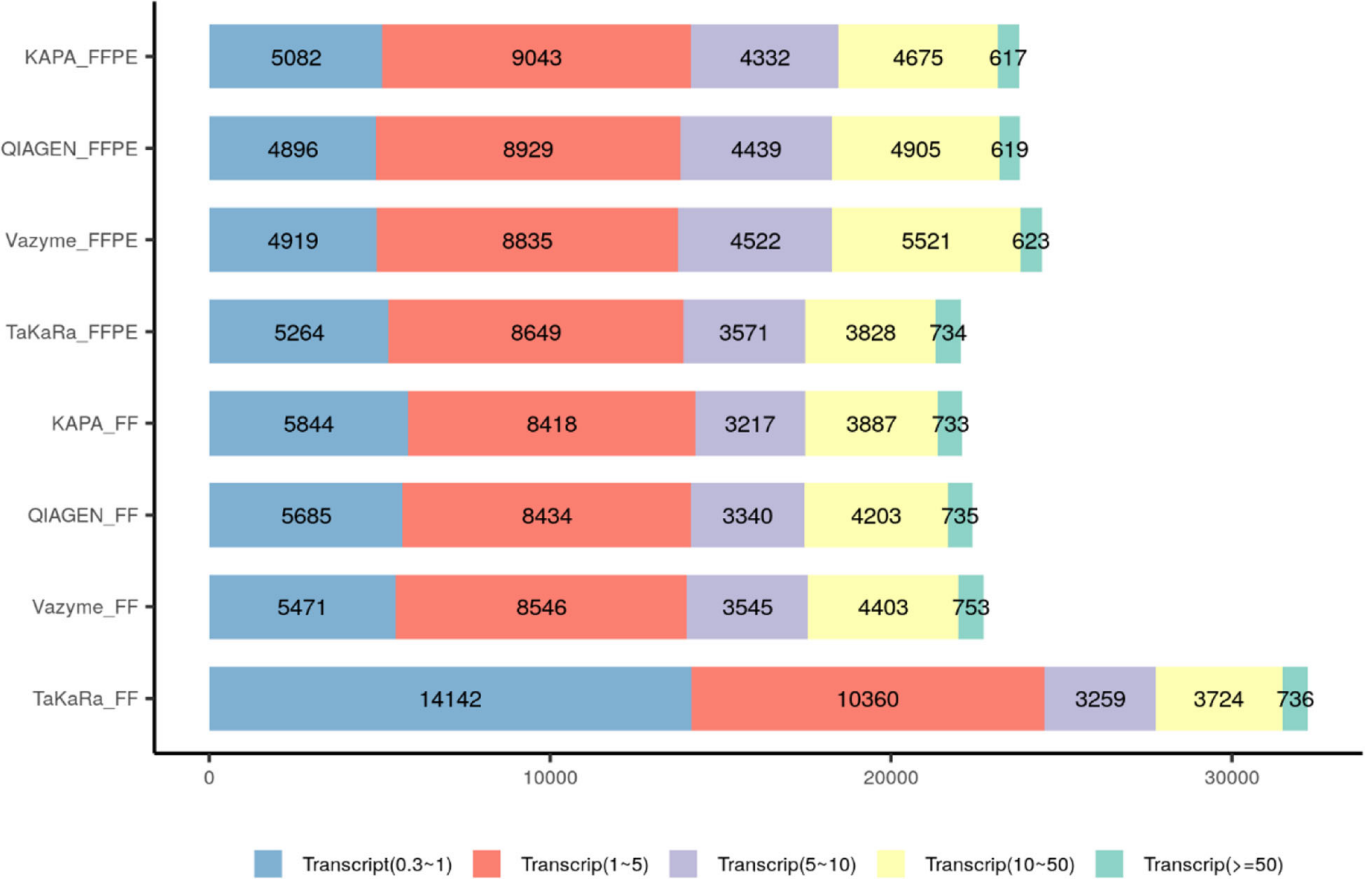
The distribution of transcripts of four RNA-seq kits with paired FFPE and FF samples. For FF RNA from GM 12878 cell line, more low-expressed transcripts were detected in the library of TaKaRa with only 10 ng total RNA input. For FFPE RNA from GM 12878 cell line, similar transcripts were detected while the input RNA of TaKaRa kit was 10 ng and it of the others was 100 ng

As shown in Figure 3, the total number of genes detected from the FFPE samples was similar among the four libraries. The number of genes detected in the TaKaRa library of the FF sample was more than twice as much as detected in the other libraries, even with using less input total RNA. We also used sample 13, sample 14 and sample 15 which were from native external quality assessment samples to test the four RNA-seq library preparation kits. As shown in Additional file 1: Table S1, we got the similar results to FFPE sample of GM12878.

**Fig. 4:**
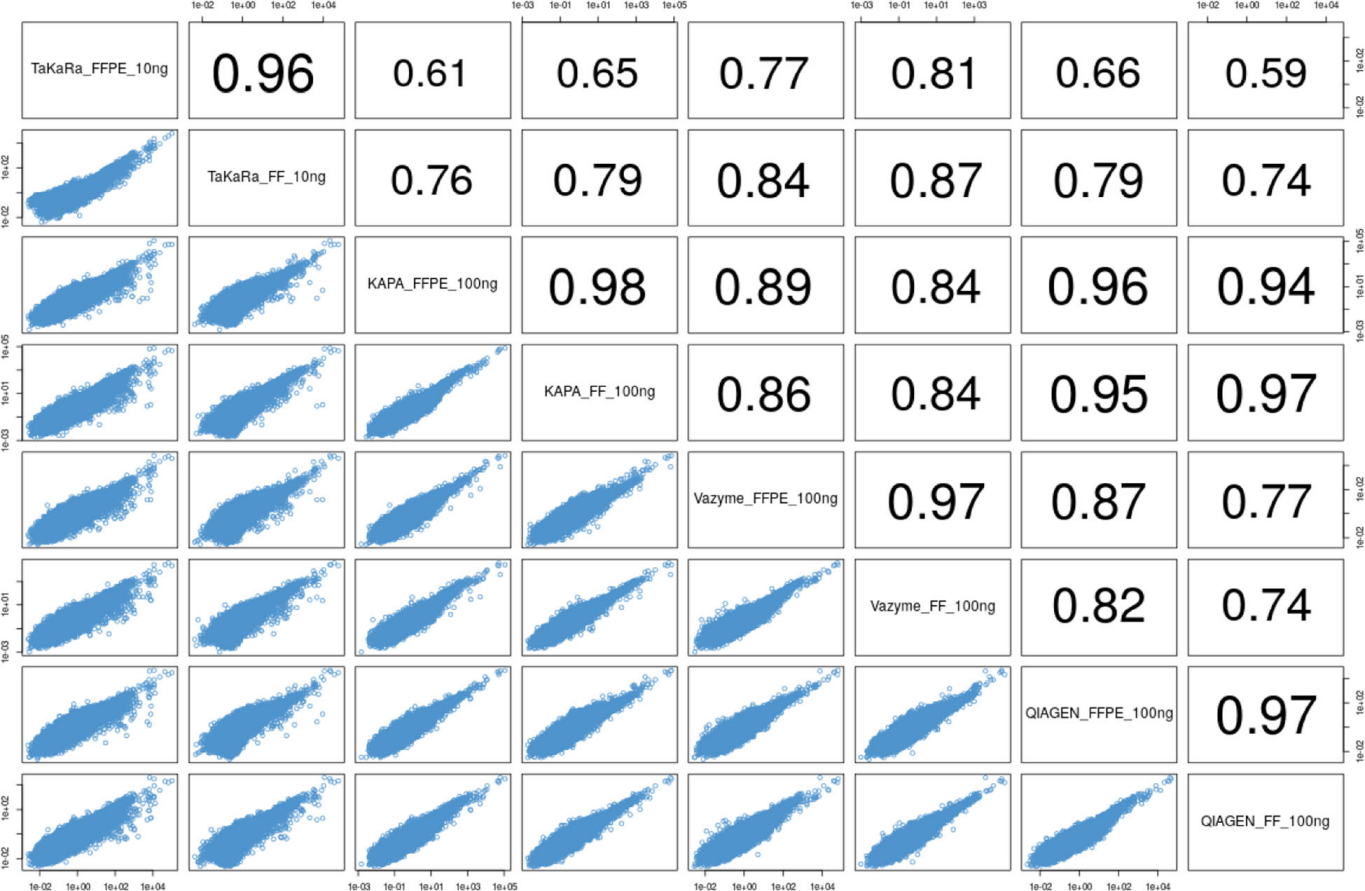
Comparison of transcripts quantification in FFPE and FF samples across four kits. High concordance in transcript quantifications were got between FF and FFPE samples using any kit. For either FFPE or FF RNA from GM 12878, the Pearson R between TaKaRa kit and the other three kits were lower and higher similarity was got among KAPA, Vazyme and QIAGEN kits

RNA-seq is an established platform for quantifying gene expression using high-quality RNA. To evaluate the gene expression performance of the FF and FFPE samples across the four kits, we compared the consistency of transcript quantification from matched pairs of FF and FFPE samples using the same kit (Figure 4). The results showed high concordance in transcript quantifications between FF and FFPE samples using the same kit (R _(FF vs FFPE)_ = 0.96 for the TaKaRa kit, R _(FF vs FFPE)_ = 0.97 for the Vazyme and QIAGEN kits, R _(FF vs FFPE)_ = 0.98 for the KAPA kit). In addition, we compared the consistency of FF (or FFPE) samples between different kits. The consistency among the KAPA, Vazyme and QIAGEN kits was higher than that of the four kits. Among the four kits, KAPA and QIAGEN showed the highest consistency, not only for FF samples (R _(KAPA vs. QIAGEN)_ = 0.97) but also for FFPE samples (R _(KAPA vs. QIAGEN)_ = 0.96). The gene expression using the TaKaRa kit showed a difference with the other kits, especially in the FFPE sample (R _(TaKaRa vs. KAPA)_ = 0.61, R _(TaKaRa vs. Vazyme)_ = 0.77, R _(TaKaRa vs. QIAGEN)_ = 0.66.), which might due to the different principle of rRNA depletion or the amount of input total RNA. The similar results were got from the test of samples 13, 14 and 15, showing in Additional file 1: Table S2.

To clarify the difference between the TaKaRa kit and any one of the other three kits in FFPE samples and FF samples, we chose the differential transcripts, which had more than a 50-fold difference. There were a total of 37 differential transcripts in the FF sample and 58 differential transcripts in the FFPE sample (Additional file 1: Table S3). There were 16 differential transcripts found both in the FF sample and in the FFPE sample. Most of these differential transcripts were mitochondrially encoded RNA, small nucleolar RNA, and 5S ribosomal pseudogene, all of which were noncoding RNA. Only a few transcripts were from coding RNA, such as the PET117 homolog, Karyopherin subunit alpha 7, and BolA family member 2B. The FPKMs of these transcripts in TaKaRa libraries were higher than those in other libraries, but not more than 10. These results indicate that the main difference between the TaKaRa libraries and the other three libraries was caused by noncoding residual RNA, and for the quantification of transcripts from coding RNA, there was no significant difference among the four RNA-seq libraries.

### Comparison of two bioanalysis methods with FF and FFPE samples

We evaluated the effect of bioanalysis tools on the total mapping rate, unique mapping rate, exon percentage and number of detected genes using FF samples and FFPE samples. For all the samples, there was almost no differences between HISAT and STAR on the quality data (Additional file 1: Table S4) regardless of RNA-seq preparation kits. Due to time and computer space, we used the HISAT analysis method to analyze data in our assay.

### RNA-seq library kit for degraded and lower input of total RNA from FFPE samples

**Fig. 5:**
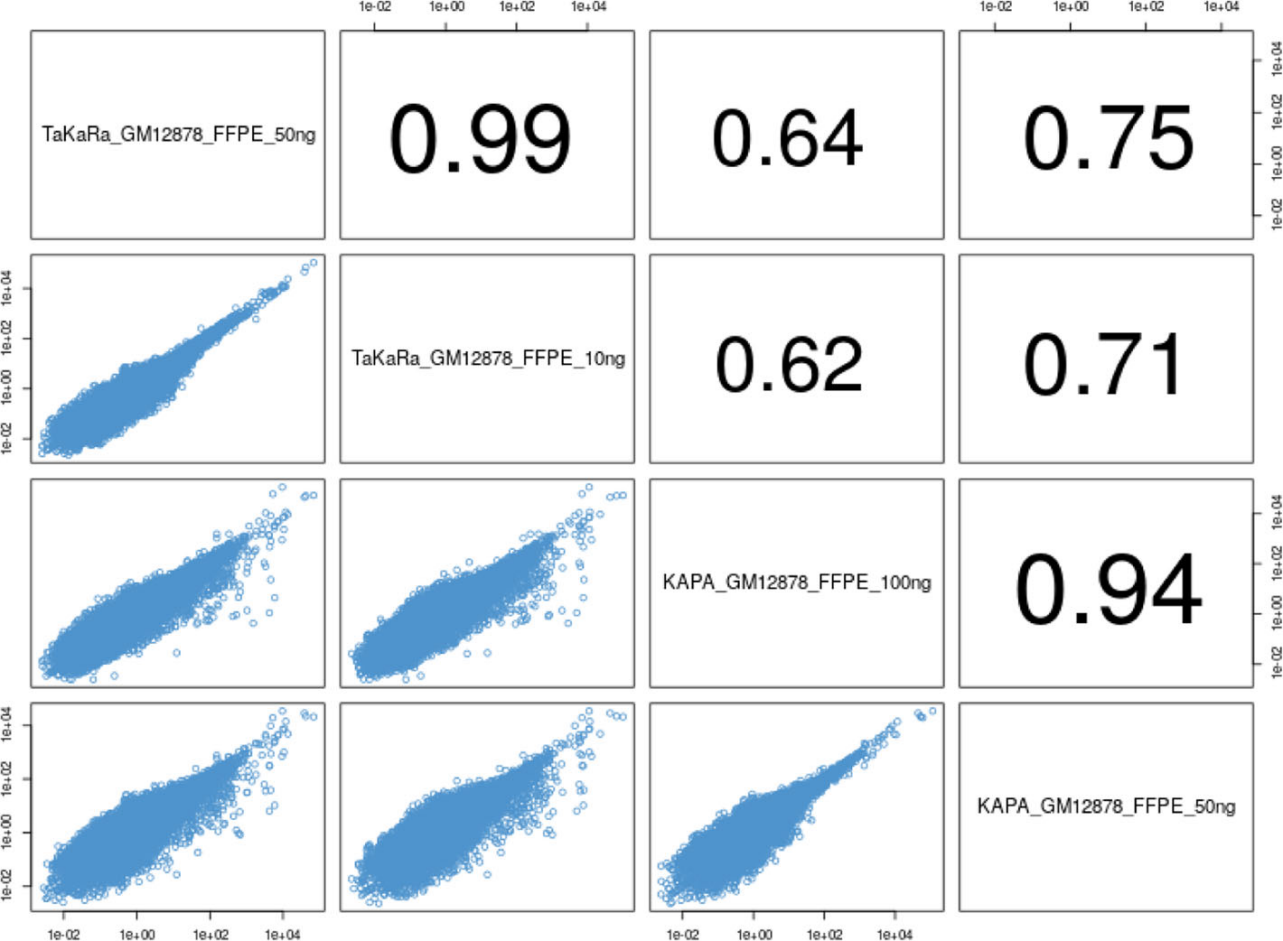
Comparison of transcripts quantification in libraries with different input of two kits. High concordance in transcript quantifications was got between 10 ng RNA input and 50 ng RNA input. For KAPA kit, although some of low-expressed transcripts were lost in the KAPA library of 50 ng RNA input, concordance in transcript quantifications was good between 100 ng and 50 ng RNA input

Many clinical studies, such as fusion detection, gene expression profiling, identification of novel transcripts and detection of alternative spicing events, want to use RNA, even though a low quality and a very low input of RNA can be extracted from clinical FFPE samples. To meet this need, we tested two kits allowing for a lower input of total RNA. The detailed results are shown in Table 2. We used the recommended cycles for each kit and obtained significantly higher library yields from the TaKaRa kit than from the KAPA kit. When raw data were down-sampled to 20 G, fewer clean data were left in the TaKaRa library because there were more reads from rRNA in its library. Although the total mapping rate in the TaKaRa library was also lower than it was in the KAPA library, exon % in the TaKaRa library was higher. A similar number of genes were detected by both kits. The correlations of transcript quantification between the two inputs and two kits are shown in Figure 5. This result demonstrated that the performance of the TaKaRa kit may be sufficient when the total RNA input is as low as 10 ng, which may be more compatible for use with RNA coming from valuable FFPE samples while reducing the depletion of samples.

**Table 2:** The performance of two RNA-seq kits allowing low total RNA input of FFPE samples

Kits	TaKaRa kit		KAPA kit	
Sample-Input	GM12878- FFPE-50 ng	GM12878- FFPE-10 ng	GM12878- FFPE-100 ng	GM12878- FFPE-50 ng
PCR cycles	13	16	15	15
Library (ng)	944.0	792.0	128.4	22.4
Total raw data (G)	20.1	21.7	35.7	24.5
Downsampled data (G)	20.0	20.0	20.0	20.0
Clean bases (G)	16.1	15.6	18.2	18.5
rRNA (%)	10.49	13.46	1.46	0.89
Q30 (%)	93.92	93.98	90.84	92.58
GC (%)	51.03	53.02	53.25	47.97
Total mapping rate (%)	92.10	91.62	96.36	97.57
Unique mapping rate (%)	80.47	79.15	80.73	87.95
Multiple mapping rate (%)	11.63	12.47	15.63	9.62
Exon (%)	61.01	64.74	53.36	46.15
Intron (%)	37.23	33.55	44.50	51.78
Intergenic (%)	1.76	1.71	2.14	2.07
Transcript (FPKM 0.3~1)	5496	5312	5240	3769
Transcript (FPKM 1~5)	9168	8680	9013	8229
Transcript (FPKM 5~10)	3612	3551	4337	4428
Transcript (FPKM10~50)	4139	3832	4664	5368
Transcript (FPKM> = 50)	730	733	621	631

### Performance of two kits with different quality of input total RNA

Another serious problem for use of clinical FFPE samples is low quality. The Agilent RNA Integrity Number (RIN) of most FFPE samples was so poor that it was not sensitive enough to evaluate the quality of RNA from degraded FFPE samples. Here, we used the reference of DV200%, the percentage of RNA fragments > 200 nucleotides, to assess FFPE RNA quality. We tested the two kits with 15 different qualities of FFPE RNA samples (Additional file 1: Figure S2). The total RNA input was 50 ng for all the samples, and the recommended PCR cycles were used for each kit. As shown in Table 3, the KAPA kit failed to construct a library for some poor quality RNA samples, or the library was insufficient to obtain more data, while all the TaKaRa libraries were successfully constructed and sequenced. Moreover, more transcripts were detected from the TaKaRa libraries than from the KAPA libraries. Similar to previous results, for all the samples when the raw data were down-sampled, fewer data were left in the TaKaRa library because residual rRNA in the TaKaRa library was much more than that of the KAPA library. The worse the quality of RNA is, the lower the percentage of exons in unique mapping reads.

**Table 3:** Comparison on the performance of the TaKaRa kit and the KAPA kit with different quality total RNA from FFPE samples

Samples	Sample13		Sample14		Sample15		Sample16		Sample17		Sample18		Sample19		Sample20		Sample21		Sample22	Sample23	Sample24	Sample25	Sample26	Sample27	R-Sample22	R-Sample23	R-Sample24	R-Sample25	R-Sample27
DV200(%)	75		77		76		61		51		52		52		46		42		2	1	11	17	26	27	2	1	11	17	27
Kits	T	K	T	K	T	K	T	K	T	K	T	K	T	K	T	K	T	K	T	T	T	T	T	T	T	T	T	T	T
PCR cycles	13	15	13	15	13	15	13	15	13	15	13	15	13	15	13	15	13	15	13	13	13	13	13	13	13	13	13	13	13
Library (ng)	115.6	48.8	448.0	31.2	242.0	17.2	91.2	9.3	472.0	24.4	280.0	38.6	62.4	5.6	200.0	13.9	155.2	4.4	48.8	83.2	216.0	98.0	161.2	344.0	8.3	14.1	58.4	20.0	123.2
Total raw data (G)	18.1	43.7	19.2	37.3	21.8	28.3	11.6	5.0	13.6	16.8	13.8	28.6	12.2	–	12.1	8.3	12.0	–	12.5	10.6	10.7	10.9	10.3	11.2	8.6	11.2	11.0	8.9	12.0
Downsampled data (G)	18.0	18.0	18.0	18.0	18.0	18.0	10.0	5.0	10.0	10.0	10.0	10.0	10.0	–	10.0	8.3	10.0	–	10.0	10.0	10.0	10.0	10.0	10.0	8.6	11.2	11.0	8.9	12.0
Clean bases (G)	15.4	15.9	15.2	16.3	14.7	16.1	6.7	4.6	7.1	9.4	7.2	9.3	7.1	–	6.0	6.6	5.0	–	3.6	5.2	7.0	6.1	6.6	6.1	2.3	5.2	8.0	4.8	6.2
rRNA (%)	5.24	5.27	4.78	2.07	10.08	5.31	20.29	1.62	14.32	0.84	14.92	1.33	12.01	–	28.24	14.96	39.42	–	47.13	35.78	15.00	38.30	24.70	41.03	47.59	34.50	11.37	31.46	39.02
Q30 (%)	94.13	91.86	94.58	92.11	93.98	92.19	91.53	92.62	92.28	93.40	92.36	93.42	89.81	–	92.12	93.00	91.51	–	77.57	83.75	90.66	87.62	91.66	92.74	73.78	82.37	89.04	84.89	90.43
GC (%)	45.27	49.86	47.05	49.16	48.40	49.70	50.79	46.64	51.58	46.71	51.02	45.99	48.20	–	48.91	46.07	52.38	–	51.23	56.66	48.76	54.56	52.66	55.30	49.17	58.32	46.99	51.75	55.23
Total mapping rate (%)	83.47	93.94	89.11	94.09	82.30	95.16	82.13	97.65	92.06	98.34	90.81	97.86	83.77	–	85.57	97.66	74.45	–	64.92	78.71	90.58	75.13	78.59	79.15	58.24	78.68	90.65	75.04	82.26
Uniquely mapping rate (%)	71.29	84.63	78.79	84.82	67.87	84.20	69.59	90.75	81.53	90.98	80.20	91.10	74.44	–	76.58	91.60	63.62	–	48.73	60.68	77.32	58.19	65.33	62.98	43.58	60.25	82.44	61.53	69.46
Multiple mapping rate (%)	12.18	9.31	10.32	9.27	14.43	10.96	12.54	6.90	10.53	7.36	10.61	6.76	9.33	–	8.99	6.06	10.83	–	16.19	18.03	13.26	16.94	13.26	16.17	14.66	18.43	8.21	13.51	12.80
Exon (%)	59.52	66.59	60.61	57.39	67.71	71.48	57.56	49.35	58.56	47.48	55.42	46.88	38.56	–	41.19	33.20	42.10	–	32.98	43.84	31.52	47.36	36.54	57.09	25.40	44.02	29.46	43.89	56.80
Intron (%)	38.31	31.86	37.68	40.92	30.50	27.40	40.80	48.93	39.86	50.68	43.08	51.40	58.92	–	57.05	64.95	55.48	–	59.33	51.77	64.92	48.06	60.59	40.88	61.77	51.68	67.10	51.15	41.47
Intergenic (%)	2.17	1.55	1.71	1.68	1.79	1.12	1.63	1.72	1.58	1.85	1.50	1.72	2.53	–	1.76	1.85	2.43	–	7.69	4.39	3.56	4.59	2.88	2.03	12.83	4.30	3.43	4.96	1.74
Transcript (FPKM 0.3~1)	5662	4724	6142	3729	5844	3118	5689	3003	6545	4496	6394	5441	5447	–	5212	2426	4885	–	2858	4560	5389	4819	5135	5755	2134	3840	4407	3636	4956
Transcript (FPKM 1~5)	9132	8294	10,045	8337	8398	6828	10,700	8659	11,841	10,819	11,566	10,839	10,888	–	10,865	8175	11,333	–	9972	10,364	11,455	10,959	11,847	9961	7588	8939	10,665	9193	9535
Transcript (FPKM 5~10)	3444	3644	3882	4359	3156	3437	3446	3627	4277	4991	3934	4192	4849	–	5003	4838	5554	–	4687	4814	5797	4918	5690	4198	4316	4544	5926	4806	4335
Transcript (FPKM10~50)	3938	4311	4146	5053	3568	4052	2539	3226	3735	4300	3132	3375	5195	–	4762	5441	4723	–	3796	4759	5538	3849	4562	4359	4175	4982	5886	4446	4572
Transcript (FPKM> = 50)	747	741	712	662	763	706	570	433	566	467	595	481	633	–	601	516	555	–	474	525	586	593	585	658	620	599	603	597	640

**Fig. 6:**
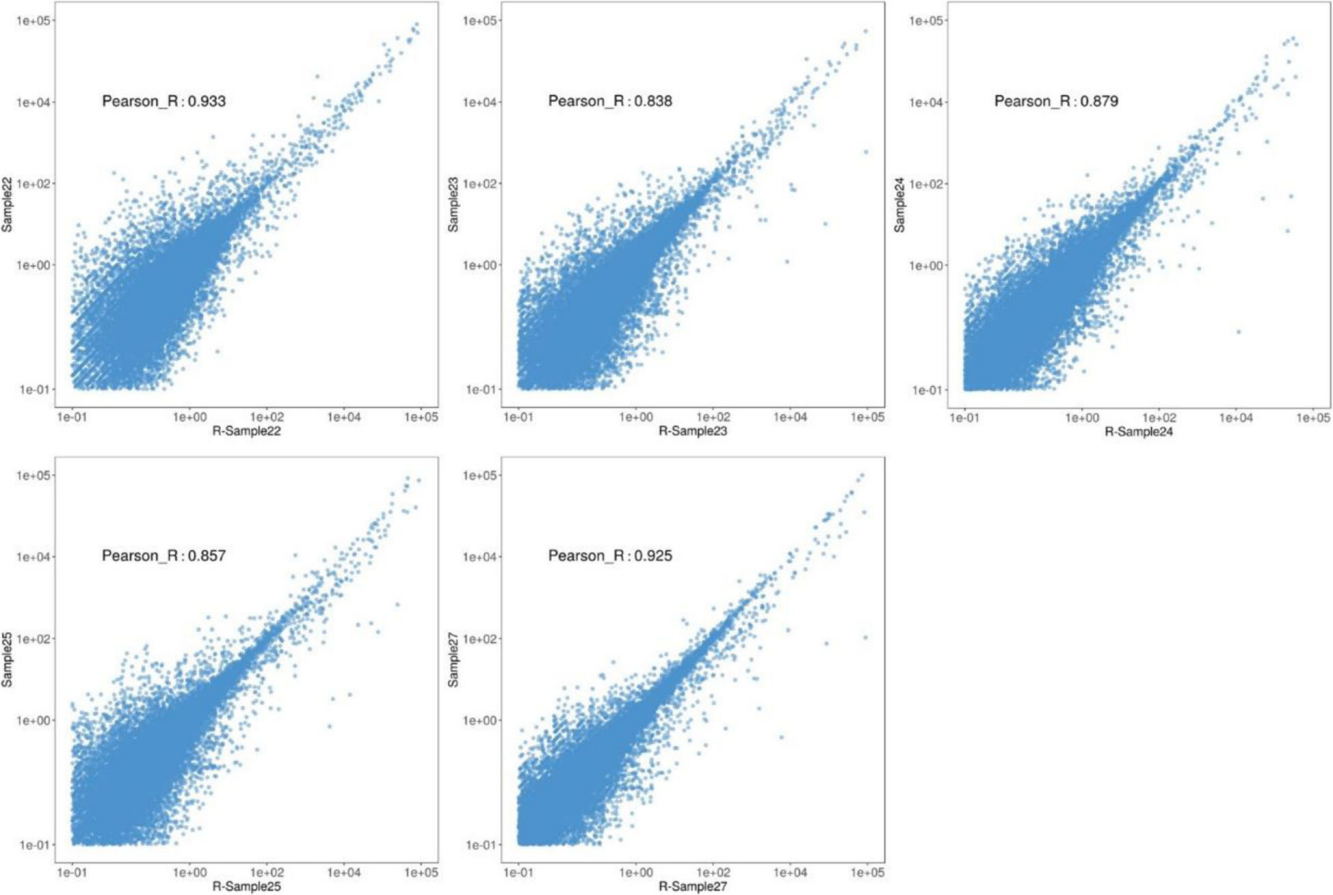
Reproducibility of transcripts quantification with low quality input total RNA. For TaKaRa kit, high concordance (R > 0.8) in transcript quantifications between the two batches with 10 ng low quality of input total RNA

To test the reproducibility of the TaKaRa kit with low quality samples, we repeated the RNA library of five FFPE samples (sample 22 to 27 except sample 26 due to insufficient total RNA). The reproducibility performance of five low-quality clinical samples was shown in Table 3. As shown in Figure 6, the results showed high concordance (R > 0.8) in transcript quantifications between the two batches. The reproducibility may be related to low quality of input total RNA.

## Discussion

RNA-seq of clinical FFPE samples could provide more important and reliable information for discovery and validation of biomarkers. Previous research [17, 18] and the results of this study also showed that FFPE RNA-seq provided reliable gene expression data, comparable to that obtained from fresh frozen tissue with the method of rRNA depletion. Standard practice in tissue fixing and paraffin-embedding has little impact on the expression analysis of RNA samples, which makes archived FFPE samples valuable and retrospective studies feasible. However, there is a limitation in the study in that we used a freshly cultured cell line and newly prepared cell FFPE blocks, and we did not compare FFPE samples with longer storage.

The difference in the principle of rRNA depletion could still result in a difference in library yield, residual rRNA, the percent of exons in unique mapping reads and transcript quantification. The KAPA kit, QIAGEN kit and Vazyme kit used the same principle of rRNA depletion, so a high concordance of transcript quantification was shown among the three kits. These three kits removed rRNA from total RNA before cDNA synthesis, using a rRNA probe to combine rRNA and then digesting rRNA by RNase H and removing the rRNA probe by DNase I. The library of the TaKaRa kit showed a difference with higher yield, residual rRNA, exon percentage and the number of detected genes, using a lower RNA input compared to the other three kits, which might result from the unique method of rRNA depletion. The workflow used in the TaKaRa kit takes advantage of a novel technology allowing removal of ribosomal cDNA (cDNA fragments originating from rRNA molecules) after cDNA synthesis using probes specific to mammalian rRNA. The specificity and number of probes could have an effect on the rRNA depletion efficiency, especially in low-quality RNA of FFPE samples, so there was higher residual rRNA in the TaKaRa library. In our results, we found that there were 9 transcripts (ENSG00000201998.1, ENSG00000200558.1, ENSG00000201321.1, ENSG00000211459.2, ENSG00000210082.2, ENSG00000207445.1, ENSG00000208892.1, ENSG00000200087.1, ENSG00000201185.1) that had very high expression (FPKM was more than 1000) in the TaKaRa libraries, while their FPKM were very low in the other kits. These transcripts included mitochondrially encoded 16S RNA, mitochondrially encoded 12S RNA, small nucleolar RNA, the 5S ribosomal pseudogene, and 5S ribosomal 9, all of which were noncoding RNAs. These transcripts detected in the TaKaRa libraries were due to low efficiency of rRNA depletion. For the same reason, we only obtained fewer clean data with the TaKaRa kit compared to the KAPA kit when using poor-quality RNA to construct libraries. The shortcoming of high residual rRNA would waste sequencing reads and increase the cost of RNA-seq.

On the other hand, the strategy of rRNA depletion after cDNA synthesis made the TaKaRa kit especially well-suited for working with very small quantities of total RNA. A similar strategy was adopted not only by TaKaRa kit but also by the Nugen kit [19]. We tried to decrease the input of total RNA, and both the TaKaRa kit and the KAPA kit showed good concordance with higher RNA input. For the TaKaRa kit, the concordance of two libraries (50 ng vs. 10 ng) was 0.99. For seriously degraded FFPE RNA, a RNA-seq library was still successfully constructed and repeated by the TaKaRa kit, but not the KAPA kit, which indicated that initial rRNA depletion from total RNA was not very effective and often leaves an insufficient amount of material for preparation of high-quality libraries.

## Conclusions

The concordance between FF and FFPE samples is excellent for any of four RNA-seq library kits. Therefore, FFPE could be used for the RNA-seq profiling with the methods of rRNA removal from total RNA. The difference between TaKaRa and the other three kits for FF and FFPE samples might be due to the different principle of rRNA removal or different input of total RNA. Both the KAPA and TaKaRa kits allowed low total RNA input and consistent transcript quantification was obtained between the lowest input and a higher input. When the quality of input total RNA was high, in which the DV 200% was more than 30%, both the KAPA and TaKaRa kits performed well. When the DV 200% of degraded RNA was less than 30%, lower quality data with lower unique mapping and lower exon percentage or failure of library construction will be evident. The TaKaRa RNA-seq library kits could be used for RNA-seq library construction of low-quality and low-quantity FFPE samples. Although the rRNA residual is a little higher, it could detect more transcripts and showed good reproducibility with low-quality and low-quantity FFPE samples.

## Methods

### GM12878 fresh cell and the preparation of cell FFPE

GM12878 cell line, which was originated from human B-lymphoblastoid cells and now often used as control sample in NGS, was obtained from Cobioer Biological Technology (Nanjing, China) and cultured using the recommended culture conditions. Briefly, GM12878 was incubated at 37 °C in an incubator (Haier, China) with 5% CO_2_ in air atmosphere, and culture media RPMI 1640 (Gibco, Cat#12633–020) contained 10% fetal bovine serum (FBS) (Gibco, Cat#10437028). About 1 × 10^7^ cells were treated with 10% neutral formalin for 1 h and then the fixed cells were collected by centrifugation of 3000 rpm for 10 min (Eppendorf, 5810R). The cells were suspended with 0.5 ml 1xPBS and then was added into 2% agarose gel. The mixture was cooled and solidified into block. The GM12878 cell block was processed according to standard formalin-fixed and paraffin-embedded (FFPE) methods [20].

### Clinical samples and ethics

There were 5 FF clinical samples (sample 1 to 5) and 22 FFPE samples (sample 6 to 27). FFPE sample 13, 14 and 15 were samples from native external quality assessment. All the 24 clinical samples (sample 1 to 27 except sample 13,14 and 15) were collected from the Cancer Hospital Chinese Academy of Medical Sciences & Peking Union Medical College. FFPE tissue slides were examined by expert pathologists including a minimum of 20% cancer cells. All these cancer samples from patients who signed the informed consent forms and were allowed to be used in other researches (Additional file 1: Table S5).

### RNA isolation and assessment of quality

GM12878 fresh cell and 5 FF samples were isolated total RNA with column purification of AllPrep DNA/RNA Mini Kit (QIAGEN, Cat#80204), and three 5um-sections of the FFPE samples were used to extract total RNA using AllPrep DNA/RNA FFPE Kit (QIAGEN, Cat#80234), both according to the manufacturer’s recommendations. We could get enough RNAs from above samples just following the detailed user guide of the kits.

The final RNA concentration was typically measured by Qubit RNA HS assay kit (Thermo Fisher Scientific, Cat#Q32855). The integrity of RNA was determined by the RNA integrity number (RIN) and DV200 (percentage of RNA fragments greater than 200 nt) with Eukaryote total RNA pico 6000 Assay of the 2100 Bioanalyzer (Agilent).

### RNA library preparation and sequencing

We used four RNA library Preparation kits including TaKaRa™ SMARTer® Stranded Total RNA-Seq Kit v2 (Takara, Tokyo, Japan, Nos. 634,413), KAPA Stranded RNA-Seq Kit with RiboErase (HMR) (KAPA, Roche Sequencing Solutions, Inc. Nos. 08098131702, 08098140702), Vaths™ Total RNA-seq (H/M/R) Library Prep Kit for illumina (Vazyme, Nanjing, China, No. NR603) and Qiagen™ Stranded Total RNA Lib Kit (Qiagen, Germany, Nos. 180,743, 180,745) kits.

We constructed libraries strictly according to the user guide of each kit and libraries prepared with the above four kits were sequenced using 150-bp paired-end runs on Illumina NGS systems (HiSeq® 2500 and Xten) after quantification by the Qubit dsDNA Assay Kit and determination of fragment length by the Agilent 2100 Bioanalyzer with the DNA 1000 Kit.

### Bioinformatic analysis

Raw data (raw reads) of fastq format were firstly processed through in-house scripts. In this step, raw reads, Q20, Q30 and GC content were calculated. Then, Trimmomatic v0.36 [21] was used to trim reads containing adapter and low quality reads from raw data. Clean data (clean reads) were obtained by removing reads mapping to rRNA reference genome using bowtie2. All the downstream analyses were based on the clean data with high quality. GENCODE GRCh37 (version 19) reference genome and gene model annotation files were downloaded from genome website directly. Then HISAT (hierarchical indexing for spliced alignment of transcripts) and STAR (Spliced Transcripts Alignment to a Reference) were both used to analyze the data of FF samples and FFPE samples. Index of the reference genome was built using HISAT2 v2.1.0 [22] or STAR v2.6.0c [15] and paired-end clean reads were aligned to the reference genome using HISAT2 v2.1.0 or STAR v2.6.0c. RSeQC v2.6.4 [23] was used to calculate how mapped reads were distributed over genome feature (exon, intron and intergenic), and nucleotide composition for each position of read. FeatureCounts v1.6.1 [24] or HTSeq v0.10.0 [25] was used to count the reads numbers mapped to each gene. FPKM, expected number of Fragments Per Kilobase of transcript sequence per Millions base pairs sequenced, considers the effect of sequencing depth and gene length for the reads count at the same time, and is currently the most commonly used method for estimating gene expression levels. And then FPKM of each gene was calculated based on the length of the gene and reads count mapped to this gene. FPKM was used to determine concordance between each kits by Pearson correlation coefficient (R).

## Supplementary information

**Additional file 1: Figure S1.** The quality of the RNA samples from GM12878 fresh cells and paired FFPE sample. **Figure S2.** The quality of the RNA samples from the fifteen clinical samples. **Table S1.** Comparison of four RNA library preparation kits for FFPE samples. **Table S2.** The consistency of transcript quantification of four RNA library preparation kits with FFPE samples. **Table S3.** The list of differentially expressed transcripts between TaKaRa and other three kits. **Table S4.** Comparison of mapping data using HISAT and STAR in FF and FFPE samples. **Table S5.** Clinical information of samples

## Data Availability

The datasets used and/or analyzed during the current study had been released and the link is https://dataview.ncbi.nlm.nih.gov/object/PRJNA555793.

## Abbreviations

cDNA: Complementary DNA
DV200%: the percentage of RNA fragments > 200 nucleotides
FF: Fresh Frozen
FFPE: Formalin-Fixed and Paraffin-Embedded
FPKM: Fragments Per Kilobase Million
FPS: Fetal Bovine Serum
HISAT: Hierarchical Indexing for Spliced Alignment of Transcripts
mRNA: Messenger RNA
PBS: Phosphate Buffered Saline
RIN: RNA Integrity Number
RNA-seq: RNA sequencing
rRNA: Ribosome RNA
STAR: Spliced Transcripts Alignment to a Reference
TOO: Tissue of Origin
