# Supplementary material for: A comparative analysis of RNA sequencing methods with ribosome RNA depletion for degraded and low-input total RNA from formalin-fixed and paraffin-embedded samples
Source: BMC Genomics. 2019 Nov 8;20:831. doi: 10.1186/s12864-019-6166-3 (PMC6842158; doi:10.1186/s12864-019-6166-3)
Supplement: Supplementary file 1 — Additional file 1: Figure S1. The quality of the RNA samples from GM12878 fresh cells and paired FFPE sample. Figure S2. The quality of the RNA samples from the fifteen clinical samples. Table S1. Comparison of four RNA library preparation kits for FFPE samples. Table S2. The consistency of transcript quantification of four RNA library preparation kits with FFPE samples. Table S3. The list of differentially expressed transcripts between TaKaRa and other three kits. Table S4. Comparison of mapping data using HISAT and STAR in FF and FFPE samples. Table S5. Clinical information of samples [file 12864_2019_6166_MOESM1_ESM.docx]

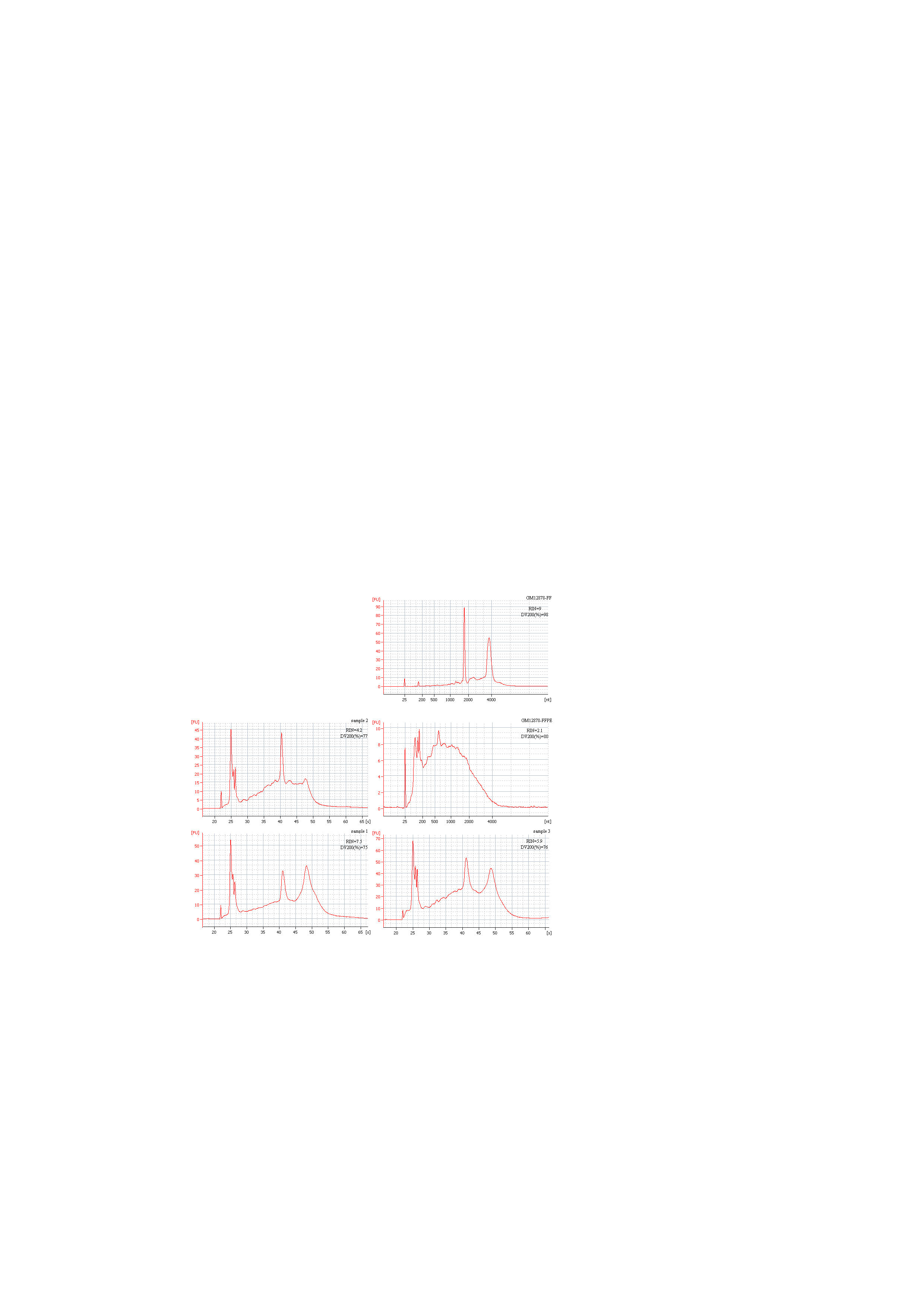

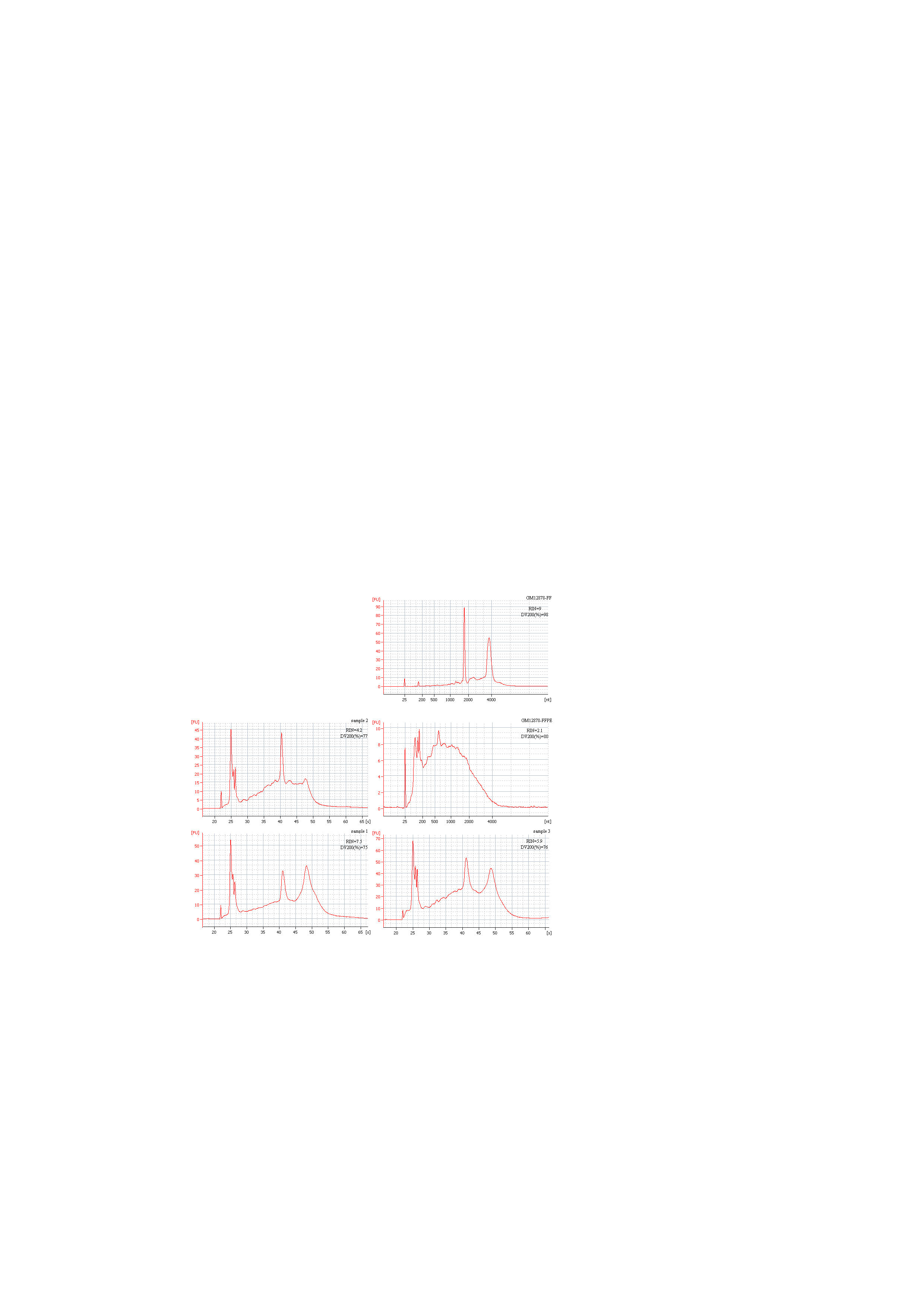


Additional file 1: Figure S1 The quality of the RNA samples from GM12878 fresh cells and paired FFPE sample.


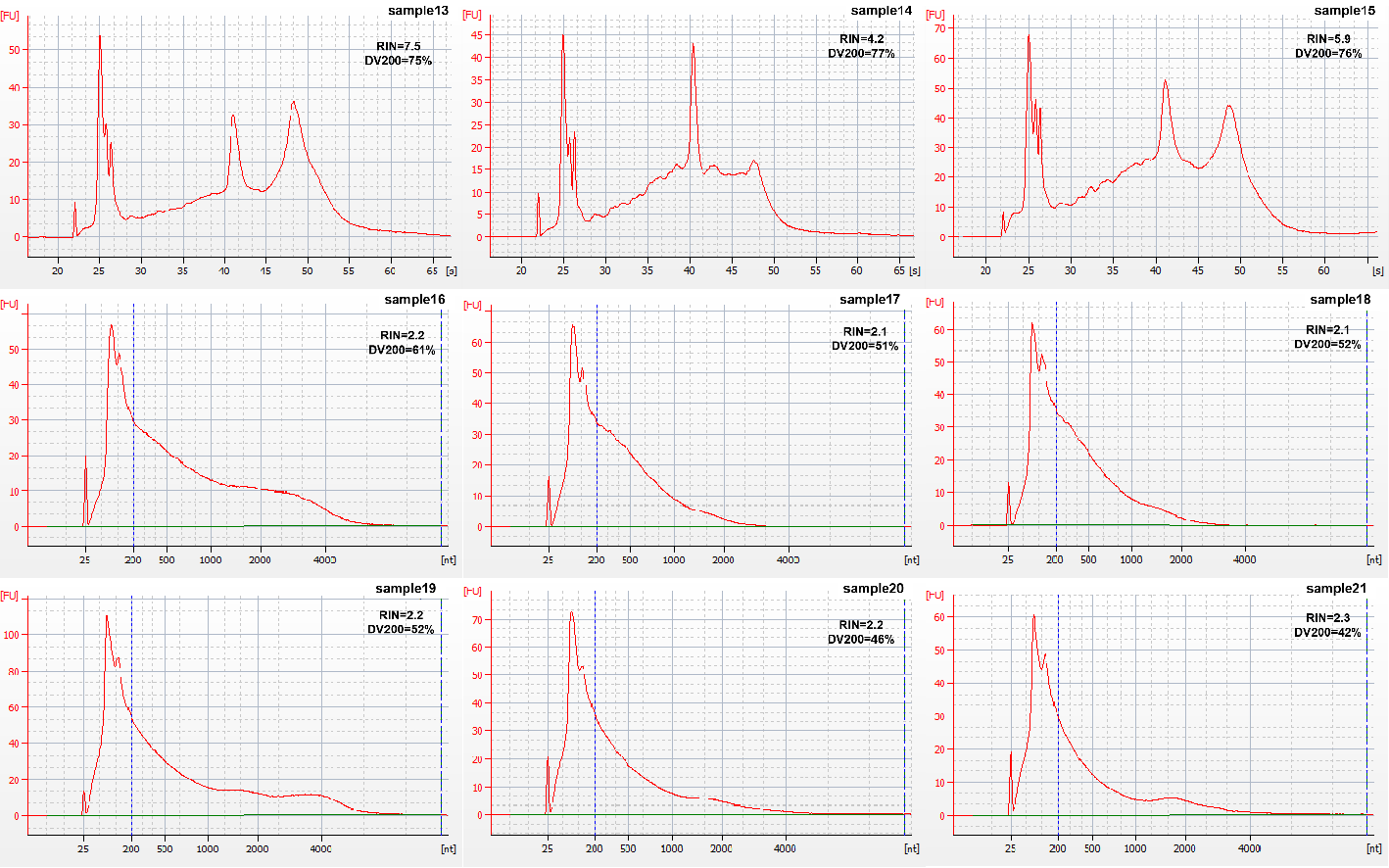


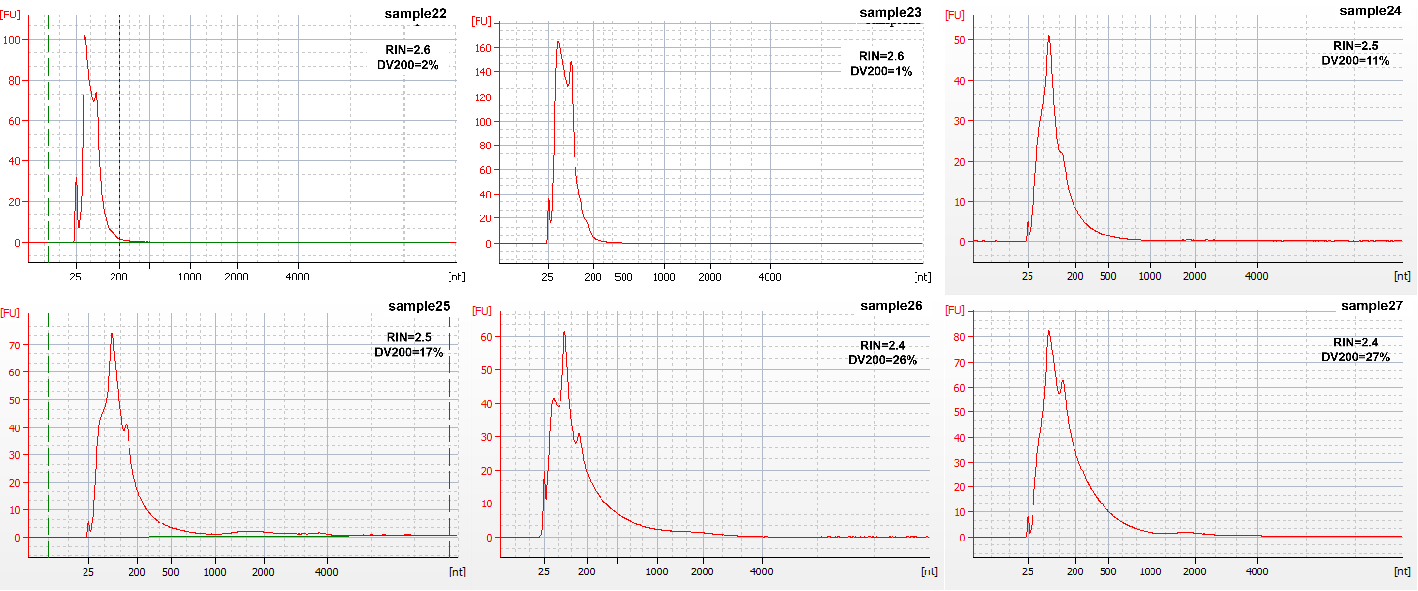


Additional file 1: Figure S2 The quality of the RNA samples from the fifteen clinical samples

Additional file 1: Table S1 Comparison of four RNA library preparation kits for FFPE samples

| Kits | KAPA | | | TaKaRa | | | Vazyme | | | QIAGEN | | |
| --- | --- | --- | --- | --- | --- | --- | --- | --- | --- | --- | --- | --- |
| Sample | sample 13 | sample 14 | sample 15 | sample 13 | sample 14 | sample 15 | sample 13 | sample 14 | sample 15 | sample 13 | sample 14 | sample 15 |
| Input total RNA (ng) | 100 | | | 50 | | | 100 | | | 100 | | |
| PCR cycles | 15 | | | 13 | | | 15 | | | 15 | | |
| Library (ng) | 48.8 | 31.2 | 17.2 | 115.6 | 448 | 242 | 124.0 | 377.5 | 200.5 | 92.4 | 118.4 | 45.2 |
| Total raw data (G) | 43.7 | 37.3 | 28.3 | 18.1 | 19.2 | 21.8 | 34.6 | 36.1 | 38.8 | 38.3 | 37.3 | 38.6 |
| Downsampled data (G) | 18.0 | 18.0 | 18.0 | 18.0 | 18.0 | 18.0 | 18.0 | 18.0 | 18.0 | 18.0 | 18.0 | 18.0 |
| Clean_bases (G) | 15.9 | 16.3 | 16.1 | 15.7 | 15.3 | 15.2 | 16.5 | 16.5 | 16.5 | 15.3 | 15.9 | 15.6 |
| rRNA (%) | 5.27 | 2.07 | 5.31 | 5.28 | 4.80 | 10.11 | 2.12 | 1.46 | 2.95 | 5.42 | 1.40 | 4.01 |
| Q30 (%) | 91.86 | 92.11 | 92.19 | 94.13 | 94.58 | 93.98 | 92.19 | 92.92 | 93.11 | 91.42 | 91.65 | 91.50 |
| GC (%) | 49.86 | 49.16 | 49.70 | 45.27 | 47.05 | 48.40 | 47.62 | 47.86 | 48.76 | 45.32 | 45.52 | 46.41 |
| Total mapping rate (%) | 93.94 | 94.09 | 95.16 | 84.03 | 89.3 | 82.97 | 93.55 | 94.15 | 94.81 | 92.82 | 93.29 | 93.59 |
| Uniquely mapping rate (%) | 84.63 | 84.82 | 84.2 | 71.05 | 78.43 | 67.64 | 84.61 | 85.72 | 83.87 | 84.78 | 85.32 | 84.64 |
| Multiple mapping rate (%) | 9.31 | 9.27 | 10.96 | 12.98 | 10.87 | 15.33 | 8.94 | 8.43 | 10.94 | 8.04 | 7.97 | 8.95 |
| Exon (%) | 66.59 | 57.39 | 71.48 | 59.52 | 60.61 | 67.71 | 62.53 | 54.08 | 69.38 | 59.23 | 51.39 | 66.08 |
| Intron (%) | 31.86 | 40.92 | 27.40 | 38.31 | 37.68 | 30.50 | 35.82 | 44.04 | 29.23 | 38.91 | 46.83 | 32.65 |
| Intergenic (%) | 1.55 | 1.68 | 1.12 | 2.17 | 1.71 | 1.79 | 1.65 | 1.88 | 1.40 | 1.87 | 1.78 | 1.27 |
| Transcript (FPKM >= 0.3) | 21710 | 22139 | 18140 | 22953 | 24925 | 21697 | 22609 | 25445 | 21376 | 23377 | 25683 | 21596 |
| Transcript (FPKM >= 1) | 16990 | 18411 | 15023 | 17318 | 18872 | 15911 | 17398 | 19784 | 15979 | 17746 | 20147 | 16255 |

Additional file 1: Table S2 The consistency of transcript quantification of four RNA library preparation kits with FFPE samples

| **sample 13** | | | | **sample 14** | | | | **sample 15** | | | |
| --- | --- | --- | --- | --- | --- | --- | --- | --- | --- | --- | --- |
| Pearson R | KAPA | TaKaRa | Vazyme | Pearson R | KAPA | TaKaRa | Vazyme | Pearson R | KAPA | TaKaRa | Vazyme |
| KAPA |  |  |  | KAPA |  |  |  | KAPA |  |  |  |
| TaKaRa | **0.866** |  |  | TaKaRa | **0.919** |  |  | TaKaRa | **0.943** |  |  |
| Vazyme | 0.977 | **0.869** |  | Vazyme | 0.959 | **0.901** |  | Vazyme | 0.964 | **0.898** |  |
| QIAGEN | 0.938 | **0.897** | 0.932 | QIAGEN | 0.95 | **0.912** | 0.928 | QIAGEN | 0.973 | **0.938** | 0.939 |

Additional file 1: Table S3 The list of differentially expressed transcripts between TaKaRa and other three kits.

| 37 differential transcripts 50 folds in FF | | | |
| --- | --- | --- | --- |
| Ensembl Gene ID | Gene Name | Ensembl Gene ID | Gene Name |
| ENSG00000121851.8 | POLR3GL | ENSG00000205609.8 | EIF3CL |
| ENSG00000169627.7 | BOLA2B | ENSG00000206596.1 | RNU1-27P |
| ENSG00000177144.5 | NUDT4P1 | ENSG00000207205.1 | RNVU1-15 |
| ENSG00000185467.7 | KPNA7 | ENSG00000207445.1 | SNORD15B |
| ENSG00000185847.3 | RP1-46F2.2 | ENSG00000208892.1 | SNORA49 |
| ENSG00000186141.4 | POLR3C | ENSG00000209082.1 | MT-TL1 |
| ENSG00000198406.6 | BZW1P2 | ENSG00000210049.1 | MT-TF |
| ENSG00000201098.1 | RNY1 | ENSG00000210077.1 | MT-TV |
| ENSG00000202354.1 | RNY3 | ENSG00000210082.2 | MT-RNR2 |
| ENSG00000202515.1 | VTRNA1-3 | ENSG00000210127.1 | MT-TA |
| ENSG00000211459.2 | MT-RNR1 | ENSG00000228519.3 | RP11-298C3.2 |
| ENSG00000214832.4 | UPF3AP2 | ENSG00000231043.2 | AC007238.1 |
| ENSG00000224722.3 | PRNCR1 | ENSG00000234589.3 | RP11-253E3.1 |
| ENSG00000224865.3 | AC009518.4 | ENSG00000235065.1 | RPL24P2 |
| ENSG00000225813.1 | AC009299.4 | ENSG00000236439.3 | RP11-175B9.3 |
| ENSG00000237649.3 | KIFC1 | ENSG00000264940.2 | SNORD3C |
| ENSG00000249784.1 | SCARNA22 | ENSG00000269028.2 | MTRNR2L12 |
| ENSG00000258628.1 | RP11-492D6.3 | ENSG00000271043.1 | MTRNR2L2 |
| ENSG00000260035.1 | CTD-2651B20.6 |  |  |
| 58 differential transcripts 50 folds in FFPE | | | |
| Ensembl Gene ID | Gene Name | Ensembl Gene ID | Gene Name |
| ENSG00000102390.6 | PBDC1 | ENSG00000202515.1 | VTRNA1-3 |
| ENSG00000121851.8 | POLR3GL | ENSG00000202538.1 | RNU4-2 |
| ENSG00000177144.5 | NUDT4P1 | ENSG00000206588.1 | RNU1-28P |
| ENSG00000185825.11 | BCAP31 | ENSG00000206596.1 | RNU1-27P |
| ENSG00000196533.6 | C1orf186 | ENSG00000206630.1 | SNORD60 |
| ENSG00000198406.6 | BZW1P2 | ENSG00000206903.1 | SNORA24 |
| ENSG00000199415.1 | RNA5SP370 | ENSG00000207093.1 | SNORD116-8 |
| ENSG00000199994.1 | RNA5SP145 | ENSG00000207174.1 | SNORD116-15 |
| ENSG00000200558.1 | RNA5SP429 | ENSG00000207263.1 | SNORD116-16 |
| ENSG00000200741.1 | RNA5SP161 | ENSG00000207279.1 | SNORD116-24 |
| ENSG00000200785.1 | SNORD8 | ENSG00000207344.1 | SNORA22 |
| ENSG00000200795.1 | RNU4-1 | ENSG00000207392.1 | SNORA20 |
| ENSG00000201098.1 | RNY1 | ENSG00000207418.1 | RNVU1-7 |
| ENSG00000201321.1 | RNA5S9 | ENSG00000207445.1 | SNORD15B |
| ENSG00000201998.1 | SNORA23 | ENSG00000208892.1 | SNORA49 |
| ENSG00000202354.1 | RNY3 | ENSG00000209082.1 | MT-TL1 |
| ENSG00000210082.2 | MT-RNR2 | ENSG00000236618.2 | PITPNA-AS1 |
| ENSG00000211459.2 | MT-RNR1 | ENSG00000239776.2 | AC079949.1 |
| ENSG00000224722.3 | PRNCR1 | ENSG00000241781.2 | AL161626.1 |
| ENSG00000228519.3 | RP11-298C3.2 | ENSG00000249784.1 | SCARNA22 |
| ENSG00000232702.2 | RP3-437C15.1 | ENSG00000251869.1 | SCARNA23 |
| ENSG00000232838.3 | PET117 | ENSG00000252316.1 | RNY4 |
| ENSG00000233891.3 | AC007131.2 | ENSG00000252947.1 | SCARNA1 |
| ENSG00000234589.3 | RP11-253E3.1 | ENSG00000260035.1 | CTD-2651B20.6 |
| ENSG00000234964.3 | FABP5P7 | ENSG00000263563.1 | UBBP4 |
| ENSG00000253007.2 | SNORA76 | ENSG00000264940.2 | SNORD3C |
| ENSG00000253190.2 | AC084082.3 | ENSG00000266019.1 | MIR3609 |
| ENSG00000259020.2 | RP11-529H20.3 | ENSG00000266638.1 | AC129492.1 |
| ENSG00000259781.1 | RP11-673C5.1 | ENSG00000270123.2 | VTRNA2-1 |

Additional file 1: Table S4 Comparison of mapping data using HISAT and STAR in FF and FFPE samples.

| samples | | RIN | | DV200% | Total mapping rate | | Unique mapping rate | | Multiple mapping rate | | exon% | | FPKM>=0.3 | | FPKM>=1 | |
| --- | --- | --- | --- | --- | --- | --- | --- | --- | --- | --- | --- | --- | --- | --- | --- | --- |
|  |  |  |  |  | HISAT | STAR | HISAT | STAR | HISAT | STAR | HISAT | STAR | HISAT | STAR | HISAT | STAR |
| FFPE-  GM12878 | GM-Kapa | | 2.1 | 80% | 96.3% | 84.7% | 80.9% | 67.6% | 15.4% | 17.1% | 53.4 | 52.7 | 23749 | 23370 | 18667 | 18434 |
|  | GM-Takara | |  |  | 91.6% | 82.2% | 79.3% | 69.9% | 12.3% | 12.3% | 64.8 | 63.3 | 22046 | 21981 | 16782 | 16845 |
|  | GM-Vaths | |  |  | 97.4% | 84.3% | 85.5% | 72.6% | 11.8% | 11.8% | 48.5 | 47.7 | 23788 | 23300 | 18892 | 18598 |
|  | GM-Qiagen | |  |  | 95.4% | 84.2% | 84.6% | 72.1% | 10.8% | 12.2% | 45.0 | 43.5 | 24420 | 23976 | 19501 | 19174 |
| FF-  GM12878 | GM-Kapa | | 9 | 98% | 96.4% | 84.7% | 79.1% | 64.4% | 17.3% | 20.3% | 75.1 | 74.1 | 22099 | 21642 | 16255 | 15884 |
|  | GM-Takara | |  |  | 93.9% | 83.4% | 80.6% | 68.6% | 13.3% | 14.8% | 70.3 | 69.0 | 32221 | 31652 | 18079 | 18015 |
|  | GM-Vaths | |  |  | 97.5% | 84.4% | 84.5% | 71.5% | 13.0% | 13.0% | 70.4 | 69.6 | 22397 | 21953 | 16712 | 16380 |
|  | GM-Qiagen | |  |  | 94.8% | 83.8% | 81.7% | 68.4% | 13.2% | 15.4% | 67.3 | 66.2 | 22718 | 22350 | 17247 | 16922 |
| FF | Sample1 | 2.4 | | 64 | 95.9% | 82.4% | 77.7% | 66.0% | 18.3% | 16.4% | 64.7 | 63.3 | 24463 | 25108 | 18532 | 19323 |
|  | Sample2 | 4.7 | | 75 | 97.4% | 87.4% | 82.8% | 73.2% | 14.6% | 14.2% | 74.9 | 74.2 | 22728 | 23115 | 16266 | 16897 |
|  | Sample3 | 2.3 | | 29 | 93.4% | 91.9% | 78.8% | 74.2% | 14.6% | 17.7% | 62.3 | 61.5 | 24889 | 25319 | 18123 | 18809 |
|  | Sample4 | 2 | | 49 | 88.2% | 88.7% | 72.9% | 73.3% | 15.3% | 15.4% | 67.6 | 67.7 | 21943 | 22749 | 16738 | 17655 |
|  | Sample5 | 2 | | 59 | 93.0% | 79.9% | 77.1% | 67.0% | 16.0% | 12.9% | 54.7 | 54.1 | 28596 | 26007 | 19806 | 21122 |
| FFPE | Sample6 | 1.2 | | 45 | 75.5% | 77.8% | 60.4% | 64.9% | 15.1% | 12.9% | 35.0 | 33.2 | 23911 | 25082 | 19161 | 20400 |
|  | Sample7 | 2.2 | | 50 | 89.4% | 89.1% | 76.8% | 77.0% | 12.7% | 12.1% | 47.6 | 47.6 | 25954 | 26567 | 19654 | 20522 |
|  | Sample8 | 2 | | 56 | 89.6% | 82.9% | 72.0% | 66.1% | 17.7% | 16.9% | 45.3 | 43.9 | 25983 | 26317 | 20371 | 21081 |
|  | Sample9 | 2.1 | | 44 | 90.6% | 89.0% | 76.9% | 75.6% | 13.7% | 13.4% | 50.0 | 50.0 | 29131 | 29748 | 20812 | 21734 |
|  | Sample10 | 2 | | 47 | 94.0% | 93.4% | 83.4% | 83.2% | 10.6% | 10.2% | 41.4 | 41.4 | 27342 | 27853 | 20349 | 21163 |
|  | Sample11 | 2.3 | | 57 | 83.2% | 83.6% | 72.0% | 73.5% | 11.1% | 10.1% | 42.6 | 42.9 | 25902 | 26462 | 19861 | 20719 |
|  | Sample12 | 2.5 | | 61 | 85.1% | 80.7% | 66.7% | 62.6% | 18.4% | 18.0% | 52.2 | 51.7 | 23050 | 23931 | 17982 | 18939 |

Additional file 1: Table S5 Clinical information of samples

| Sample types | Sample name | | Gender | Age | Cancer |
| --- | --- | --- | --- | --- | --- |
| FF | Sample1 | Female | | 61 | colon cancer |
|  | Sample2 | Female | | 54 | ovarian cancer |
|  | Sample3 | Male | | 63 | prostate cancer |
|  | Sample4 | Female | | 71 | lung cancer |
|  | Sample5 | Female | | 46 | cervical cancer |
| FFPE | Sample6 | Male | | 40 | lung cancer |
|  | Sample7 | Male | | 49 | lung cancer |
|  | Sample8 | Male | | 47 | lung cancer |
|  | Sample9 | Female | | 43 | gastric carcinoma |
|  | Sample10 | Female | | 47 | thymic carcinoma |
|  | Sample11 | Female | | 63 | lung cancer |
|  | Sample12 | Male | | 61 | renal carcinoma |
|  | Sample13* | — | | — | — |
|  | Sample14* | — | | — | — |
|  | Sample15* | — | | — | — |
|  | Sample16 | Female | | 69 | liver cancer |
|  | Sample17 | Male | | — | liver cancer |
|  | Sample18 | Female | | — | liver cancer |
|  | Sample19 | Male | | 42 | liver cancer |
|  | Sample20 | Male | | 51 | liver cancer |
|  | Sample21 | Female | | 38 | liver cancer |
|  | Sample22 | Female | | 48 | colon cancer |
|  | Sample23 | Male | | 38 | liver cancer |
|  | Sample24 | Male | | 49 | esophageal carcinoma |
|  | Sample25 | Male | | 60 | liver cancer |
|  | Sample26 | Female | | 33 | liver cancer |
|  | Sample27 | Male | | 26 | rectal cancer |

***** samples from native external quality assessment
